# Beyond Cocrystals: Hierarchical Functional Assemblies via Noncovalent Synthesis

**DOI:** 10.1002/advs.202522508

**Published:** 2026-01-27

**Authors:** Ya‐Nan Zhu, Jin Feng, Ying‐Xin Ma, Hong‐Tao Lin, Li‐Wei Xie, Qi‐Liang Peng, Xue‐Dong Wang

**Affiliations:** ^1^ State Key Laboratory of Bioinspired Interfacial Materials Science Institute of Functional Nano & Soft Materials (FUNSOM) Soochow University Suzhou China; ^2^ Department of Radiotherapy and Oncology The Second Affiliated Hospital of Soochow University Suzhou China; ^3^ Institute of Chemistry and Chemical Engineering Shandong University of Technology Zibo Shandong P. R. China

**Keywords:** hierarchical structures, non‐covalent interactions, optoelectronic applications, organic cocrystals, sequential self‐assembly

## Abstract

Cocrystals are solid crystals formed by the combination of multiple components through non‐covalent interactions (NCIs). Despite their diverse structures and tunable optoelectronic properties, they cannot meet the demand for multi‐functionalization of optoelectronic devices. Therefore, a series of strategies is summarized to construct organic hierarchical structures (OHSs) by self‐assembly of NCIs using organic cocrystals as base building blocks. Sequential nucleation of cocrystals has been achieved by regulating the strength of NCIs to further synthesize complex OHSs. This review explores the fundamental factors affecting the physicochemical properties of cocrystals, such as molecular arrangement, the distance and orientation of intermolecular interactions, etc. Additionally, the self‐assembly process of synthetic OHSs driven by single & multiple NCIs is analyzed, along with the applications of these structures in optoelectronics. Finally, urgent issues on preparing OHSs are raised, and an outlook on possible future opportunities is provided.

## Graphical abstract

Non‐covalent interactions (NCIs) drive the formation of organic cocrystals with diverse structures and tunable optoelectronic properties. This review explores the essential factors governing these properties, highlighting how the sequential nucleation of cocrystals leads to the self‐assembly of organic hierarchical structures (OHSs). Finally, various application‐oriented structures are presented, and challenges in preparing OHSs are discussed.

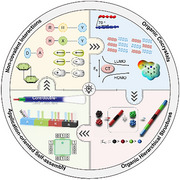

## 1 Introduction

Since 1844, the first reported cocrystal (1:1) of benzoquinone 1,4‐Benzoquinone and Hydroquinone initiated the study of organic cocrystals. Further to the discovery of the high electrical conductivity of tetrathiafulvalene‐7,7,8,8‐tetracyanoquinodimethane (TTF‐TCNQ) cocrystals in 1973 [1], attracted much attention in the field of optoelectronics. In 1995, Kochi's team demonstrated that charge transfer excitons generated in 1,2,4,5‐benzeneenetetranitrile (TCNB)‐based cocrystals can be relaxed to free carriers [2], suggesting that these cocrystals are ideal candidates for photovoltaic conversion. With the in‐depth study of the crystalline structure of organic molecules and the development of computational chemistry and molecular simulation techniques [3, 4], the key role of NCIs in cocrystal formation is gradually revealed. For example, the assembly of organic ionic cocrystals using silver nanoclusters and organic macrocycles exhibits excellent circularly polarized optical activity [5].

During the formation of organic cocrystals, NCIs mainly contain hydrogen bond (HB) [6, 7, 8], Halogen bond (HaB) [7, 9], *π–π* interaction [10], charge transfer interaction(CT) [11, 12, 13, 14], van der Waals forces(VDW), and so on. Although they are not as strong as covalent bonds, they can maintain the structural stability of a substance to a certain extent. The assembly of organic molecules into highly ordered crystal structures is driven by dynamic, reversible intermolecular interactions. In pharmaceutical research, the physicochemical properties of drugs can be improved without altering the structure of the active pharmaceutical ingredient (API) by introducing HB and *π–π* interaction, such as the drug‐drug cocrystal of favipiravir (FPV) with salicylamide (SAA), which exhibited a dual improvement in solubility and permeability [15]. In addition, charge transfer (CT) interaction, which leads to a decrease in band energy between the electron donor and acceptor, promotes electron excitation and transfer, providing more opportunities for the development of high‐performance optoelectronic (photothermal conversion [16], two‐photon excitation [17], thermally‐activated delayed fluorescence(TADF) [18], vapochromism [12], room‐temperature phosphorescence [18]). Whereas the weak interactions of VDW are widespread between all molecules regardless of their charge or polarity, and VDW are not directional. Together, these noncovalent interactions determine the formation, structure, and physicochemical properties of cocrystals.

However, against the backdrop of growing demand for complex multifunctional devices, organic hierarchical structures (OHSs) are attracting immense attention for integrating a variety of materials and functions compared to single cocrystals. In our definition of hierarchical structure [19, 20, 21, 22], hierarchical structure is a multilevel organization that first assembles basic molecular units into ordered secondary structures via NCIs, and then uses the secondary structures as building blocks to form more complex multifunctional superstructures at the next level(s). These properties, such as multicolor emission in multi‐blocks [23], multichannel output in branches [24, 25, 26], and increased carrier density [27] at the core/shell interface, create more possibilities for the development of optoelectronic devices. The preparation of OHSs follows the advantages of low‐cost processing and simple operation of organic cocrystals [28, 29], but challenges remain in large‐scale preparation due to the uncertainties in the kinetic process and the high energy barrier that is difficult to surmount in the thermodynamic process.

In summary, we propose a hierarchical self‐assembly strategy that integrates the advantages of cocrystals and heterostructures. Based on cocrystals, organic hierarchical structures with accurate spatial organization are rationally designed to meet various application requirements by precisely regulating NCIs. NCIs are like invisible bridges, skillfully linking cocrystals and OHSs, preserving the properties of each component while enabling them to create new synergies in their interactions. In this review, we investigate the relationship between NCIs and the physicochemical properties of cocrystals. Selectively introduced NCIs are highlighted for diversity and flexibility in hierarchical self‐assembly processes based on organic cocrystals. In addition, we demonstrate cocrystals and OHSs specifically engineered for diverse application requirements, which exhibit tailored optoelectronic properties.

## 2 Hierarchical Self‐Assembly Strategy

**FIGURE 1:**
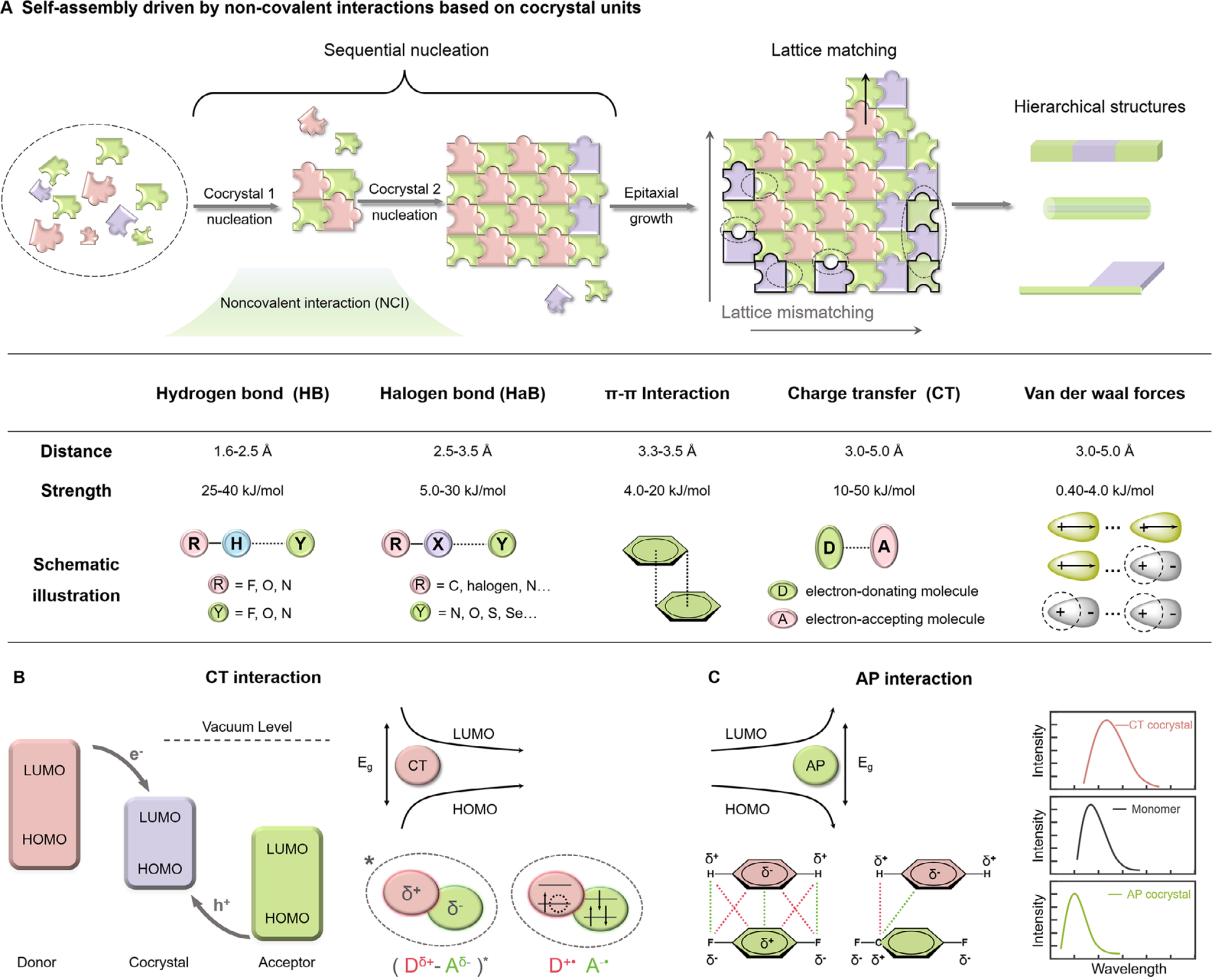
Noncovalent interactions (NCIs) driving organic hierarchical self‐assembly. (A) Schematic diagram of organic molecules sequentially nucleating to form cocrystals and epitaxially growing to form organic hierarchical structures (OHSs), and schematic diagram of NCIs driving self‐assembly. (B) Charge transfer (CT) interaction. Schematic diagram of electron donors, acceptors, and HOMO‐LUMO forming CT cocrystals; energy gap increases after CT cocrystal formation; schematic diagram of partial & complete CT states. (C) Arene‐perfluoroarene (AP) interaction. Energy gap decreases after AP cocrystal formation; diagram of the wavelengths of CT, AP cocrystals, and monomers. Reproduced with permission [49] Copyright 2024, Elsevier.

In order to construct an organic hierarchical structure (OHS), one starts with fundamental building blocks (molecules)making up that structure. These building blocks are then sequentially combined into different cocrystals, depending on the strength of non‐covalent interactions(NCIs). Besides, there should be good structural and chemical compatibility between each building block for better lattice matching. As illustrated in Figure 1A, the degree of flexibility with which the green blocks can be put back together with the red and purple blocks, respectively, represents the strength of the NCIs between them. Higher bonded blocks(green & red) preferentially achieve nucleation and self‐assembly into cocrystals. Next, weakly bonded blocks (green & purple) nucleate on the former, and eventually grow epitaxially into OHSs. Another issue to be aware of is that blocks, when spliced together, can cause varying degrees of lattice mismatch due to differences in lattice parameters, resulting in them growing only along one or a few specific crystal planes.

### 2.1 Noncovalent Interactions (NCIs)

NCIs are vital in controlling the structural integrity and stability of cocrystals and OHSs. Hydrogen bond (HB) [30] consists of a proton donor and a proton acceptor (usually electronegative atoms, such as N, O, F, etc.), which are directional and saturated, moderately strong and reversible, widely used in drug design, nucleic acid base pairing [31]. Halogen bond (HaB) (R—X…Y)is composed of an electrophilic halogen bond donor (usually an electrophilic σ‐hole or π‐hole on the halogen atom [32]) and a nucleophilic HaB acceptor. In the same period, halogen atoms are more electronegative and have a stronger ability to attract electrons. In other words, the HaB donor is the electron acceptor, and the HaB acceptor is the electron donor. The strength of HaB is determined by the size of the σ/π‐hole on the backside of the halogen atom [33]. Halogen atoms with lower electronegativity and higher polarizability are more likely to form halogen bonds (F<Cl<Br<I<At). HaB exhibits a certain directionality: The X atom in R‐X (where X is a halogen) typically engages through its axial σ‐hole region, while the HaB acceptor Y tends to interact with the lateral regions of covalently bonded halides or other electron‐rich entities [32]. This high directionality enables HaB to drive the assembly of diverse crystalline architectures, such as 1D rod [34], 2D sheet [34, 35], and beyond.

*π–π* interaction occurs mainly between aromatic rings, including face‐to‐face stacking (sandwich‐type), offset stacking, and edge‐to‐face stacking (T‐type) [36, 37, 38]. It can shorten the molecular spacing, promote dense molecular stacking, facilitate the transport of electrons and holes, and improve the optoelectronic properties of the material [24, 37]. Van der Waals (VDW) forces comprise three components: *Keesom interactions* (orientation forces between permanent dipoles in polar molecules), *Debye interactions* (induction forces between permanent and induced dipoles), and *London dispersion forces* (dispersion forces, transient dipole‐dipole interactions in nonpolar molecules). Although van der Waals forces are relatively weak (0.5–3 kcal/mol [39]), they are ubiquitous in conjugated molecular systems, supramolecular assemblies [40], 2D materials [41, 42, 43], and metal‐organic interfaces.

When the electron donor (D) or electron acceptor (A) in a system becomes excited, intramolecular electronic transitions occur between molecular orbitals, resulting in the formation of D^*^A or DA^*^ [44], corresponding to the local excited (LE) state (no charge transfer occurs). In the local excited (LE) state, partial charge transfer takes place between D and A, known as the charge transfer (CT) state of (D ^δ+^‐A ^δ−^) ^*^(Figure 1B). Following the CT state formation, the system evolves into the charge‐separated (CS) state via complete charge transfer, with the resultant electron‐hole pair fully dissociating into independently mobile free carriers (D^+•^‐A^−•^) [45]. In organic photovoltaic (OPVs), the CT state is the key to achieving efficient charge separation. Moreover, in organic light‐emitting diodes (OLEDs), the LE state serves as the primary source of emission, and the CT state achieves efficient luminescence via reverse intersystem crossing (RISC). Thus, understanding and regulating the transition between LE and CT states are essential for enhancing energy conversion efficiency.

Charge transfer (CT) interaction plays a dominant role in the formation of organic cocrystals. In CT cocrystals, the highest occupied molecular orbital (HOMO) is contributed by the electron donor, while the lowest unoccupied molecular orbital (LUMO) stems from the electron acceptor. Compared to isolated D and A, the CT cocrystal exhibits a lower HOMO energy (derived from the D) and a higher LUMO energy (from the A) [46] s(Figure 1B). This indicates a reduced energy gap of the cocrystal (decreased energy of emitted photons), leading to a red shift [17, 47, 48] in the spectrum (Figure 1C). Furthermore, the narrower energy gap between D's HOMO and A's LUMO, the stronger the strength of CT interaction.

Arene‐perfluoroarene (AP) interaction belongs to a special kind of *π–π* interaction. In *π–π* interaction, the sandwich face‐to‐face geometry arises from electrostatic repulsion and attraction, whereas the offset stacking and T‐type are dominated by favorable electrostatic attraction [49]. According to the electronegativity order of H<C<F, the carbon skeleton of benzene exhibits negative character while its periphery shows positive charge. Although benzene is a nonpolar molecule, it possesses a significant quadrupole moment. In contrast, perfluorobenzene displays an inverted quadrupole relative to benzene [36]. This complementary quadrupole moment results in benzene and perfluorobenzene stacking face‐to‐face in an electrostatic attraction (with a certain crossed angle).

For other molecules with AP interactions, such as octafluoronaphthalene and aromatic hydrocarbons (naphthalene/anthracene/pyrene/perylene), the approaching molecules tend to adopt an offset‐stacked geometry to optimize the balance between electrostatic interactions and dispersion forces, thereby achieving lower overall energy (Figure 1C). Based on the above electrostatic coupling and dispersive forces, the energy gap between HOMO‐LUMO between arene and perfluoroarene becomes larger, resulting in a blue shift [50].

In summary, both AP and CT interactions lead to electron cloud redistribution. AP interaction involves polarization‐driven local adjustments (no net charge transfer), whereas the CT interaction entails a charge‐transfer‐driven global reorganization. This intrinsic distinction provides a theoretical basis for the targeted design of non‐covalent functional materials (e.g., organic semiconductors, responsive sensors).

## 3 Physicochemical Properties of Cocrystals

**FIGURE 2:**
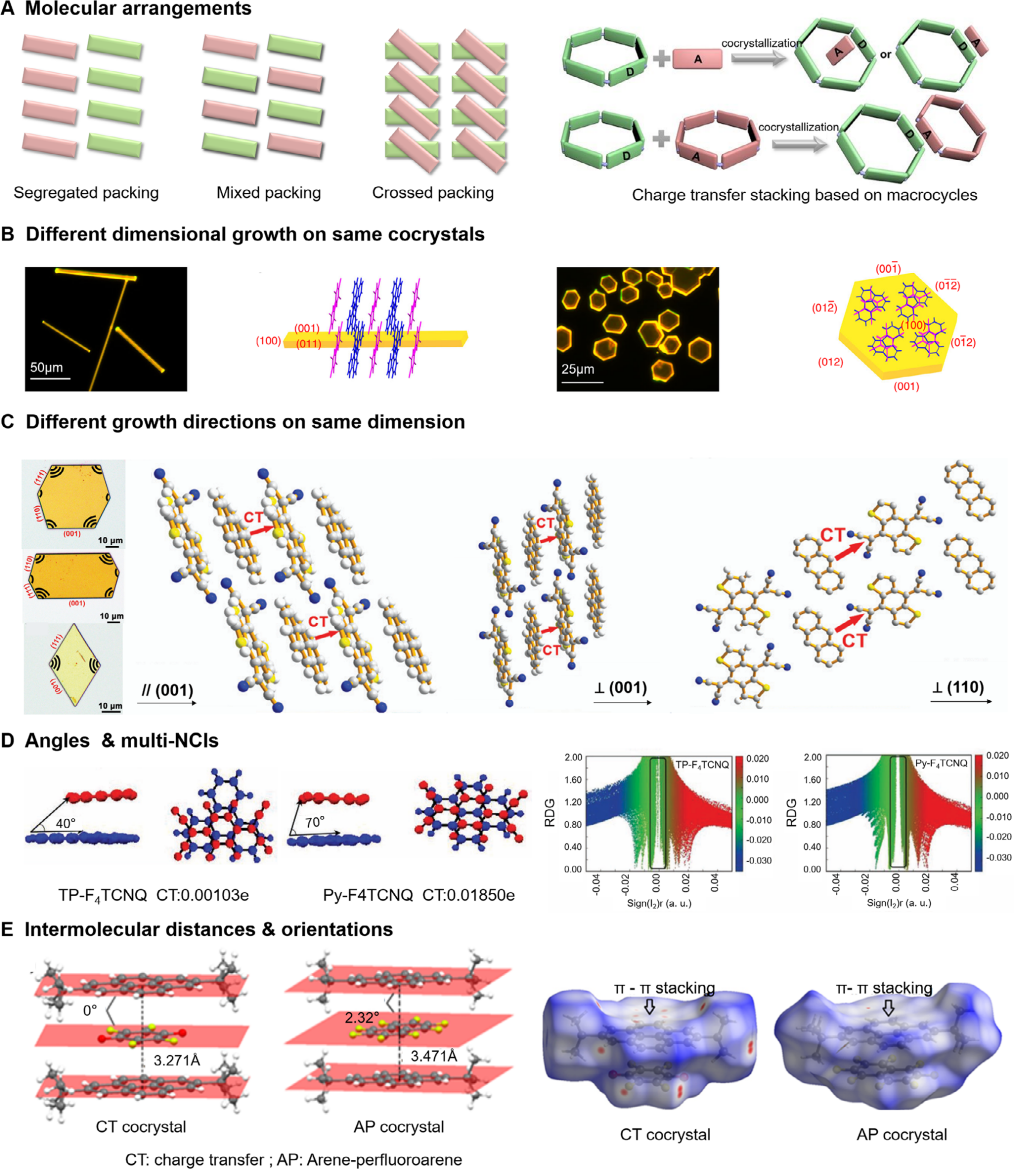
Physicochemical Properties of Cocrystals. (A) Molecular arrangements of an organic cocrystal. Reproduced with permission [66]. Copyright 2023, Wiley‐VCH GmbH. (B) Different dimensional growth on the same cocrystals. Reproduced with permission [74]. Copyright 2019, Springer Nature. (C) Different growth directions on the same dimension. Reproduced with permission [76]. Copyright Royal Society of Chemistry. (D) Angles & multi‐NCIs. Reproduced with permission [52]. Copyright 2022, Wiley‐VCH GmbH. (E) Intermolecular distances & orientations. Reproduced with permission [77]. Copyright 2019, American Chemical Society.

Compared to organic single‐component crystals, organic cocrystals exhibit superior performance in optical [51, 52, 53], thermal [54, 55, 56], electrical, magnetic [11, 13], and mechanical [54, 56, 57] properties. These enhancements are fundamentally attributed to differences in molecular arrangement, packing angles, and distances between molecules, as well as the strength of NCIs. The molecular arrangement significantly affects the charge transport properties [52, 58] and luminescence characteristics [52, 59, 60]. In organic cocrystals, there are two common types of molecular stacking: segregated stacking and mixed stacking (Figure 2A). The segregated stacking (‐DDD‐, ‐AAA‐) shows strong charge transfer characteristics [52] and high conductivity [61] due to large molecular orbital overlap [62]. Whereas the mixed stacking (‐DADADA‐) exhibits weak charge transfer properties and non‐conductivity while maintaining superior charge transport performance. For instance, pyrene‐N, N′‐bis(perfluorobutyl)‐1,7‐dicyanoperylene‐3,4:9,10‐bis(dicarboximide) cocrystal‐based field‐effect transistors (FETs) exhibit balanced electron and hole mobilities, enabling ambipolar charge transport [63].

Furthermore, crossed packing (X) effectively mitigates material anisotropy while enhancing device sensitivity [64] and stability [65]. As demonstrated by Ehud Gazit et al. [65], cocrystallization of 4,4′‐bipyridine (4,4′‐Bpy) and N‐acetyl‐_L_‐alanine (AcA) achieves H→J/X (crossed stacking) hybrid packing: J‐type aggregation reduces *π–π* overlap to suppress aggregation‐caused quenching (ACQ) with fluorescence redshift, while X‐aggregation reinforces structural rigidity to improve mechanical stability(Figure 2A). This design provides a new paradigm for co‐assembly‐regulated stacking strategies, expanding possibilities for constructing functional materials from simple molecules.

In recent years, macrocyclic compounds have been strategically employed to construct supramolecular CT cocrystals owing to their intrinsic cavity architectures and multiple non‐covalent binding sites [66]. The synergy between macrocyclic host‐guest chemistry and cocrystal engineering, where guests engage with macrocycles through either cavity encapsulation or exterior‐wall binding, offers more opportunities in optoelectronics (e.g. multicolor emission system [48, 67] and thermally activated delayed fluorescence (TADF) materials [48, 68, 69]), smart separation (vapochromic sensors for haloalkanes [70]/alkyl ketones [71] detection) and drug recognition [72].

The growth direction of organic cocrystals depends on the dominance of interaction strengths along specific crystallographic planes or directions. For example, the driving force for the 1,2‐bis(4‐pyridyl) ethylene (Bpe)‐1,3,5‐trifluoro‐2,4,6‐triiodobenzene (IFB) cocrystal is the strong *π–π* interaction along the [99] direction, leading to self‐assembly into 1D linear structures. In contrast, for the Bpe‐1,4‐diiodotetrafluorobenzene (F_4_DIB) cocrystal, N···I halogen bonding and C−F…H hydrogen bonding play dominant roles, resulting in a 2D block‐like morphology.^34^For the same cocrystal system, adjusting external conditions (e.g., precursor solution concentration or temperature) can enable growth in different dimensions. For example, the phenazine (Phz)‐chloranilic acid (H_2_ca) cocrystal (PHC) exhibits a kinetically stable 1D linear structure at low concentrations and a thermodynamically stable 2D plate‐like structure at supersaturation [73]. In the 1D self‐assembly process, *π–π* interactions act as the primary driving force along the [99] crystallographic direction, whereas in 2D self‐assembly, due to supersaturation, the growth barriers of (200) and (020) are overcome, and driven by *π–π* interaction and HB, PHC achieves a 2D growth along the [99] and [010] directions. Moreover, increasing the temperature facilitates a morphological transition from rod‐like to hexagonal plate‐like structures in the fluoranthene‐TCNB cocrystal [74]. Such directional changes in growth do not change the molecular packing mode; the nature of this shift is, in fact, the result of the modulation between metastable kinetic processes and stable thermodynamic equilibrium during cocrystal growth (Figure 2B). For co‐crystals with the same composition, different molecular distances may lead to distinct non‐covalent interactions (NCIs), resulting in varied packing patterns, which can further influence their morphology and luminescence properties [75]. For example, the co‐crystal formed by 4‐(4‐Dimethylaminostyryl)quinoline (DMAQ) and 1,4‐Diiodotetrafluorobenzene (FDIB) exhibits two different forms: one is green hexagonal M‐DFCs, and the other is yellow rhomboidal T‐DFCs. M‐DFCs adopt a segregated stacking mode driven by halogen bonding (HaB) and π–π interactions (with a distance of 3.38 Å). In contrast, T‐DFCs adopt a mixed stacking arrangement driven solely by HaB, where the face‐to‐face stacking distance between donor and acceptor molecules is too large (3.71 Å) to allow effective π–π interaction. Consequently, these two types of co‐crystals show significant differences in both emission properties and polarization direction.

Further, different growth directions in the same dimension also have an effect on the properties of organic cocrystals. For example, Anthracene (An)‐4,8‐bis(dicyanomethylene)4,8‐dihydrobenzo[1,2‐b:4,5‐b']‐dithiophene (DTTCNQ) cocrystals have hexagonal and rhombic 2D morphologies (Figure 2C). Although the dipolar transport properties can be measured in all crystal growth directions, the electronic properties undergo a change from n‐type‐dominated dipolar, balanced bipolar to p‐type‐dominated bipolar upon changing the measured orientation of the crystals [76].

The stacking angles between molecules and the number of NCIs also significantly influence the properties of cocrystals. For example, the TP‐F_4_TCNQ cocrystal exhibits highly efficient near‐infrared (NIR) emission, whereas Py‐F_4_TCNQ displays fluorescence quenching [52]. In TP‐F_4_TCNQ, the smaller counter pitch angle between molecules in face‐to‐face stacking and overlap weakens π‐conjugation, resulting in a reduced charge transfer (CT) degree (Figure 2D). Furthermore, analysis based on the reduced density gradient (RDG) and Sign(λ_2_) ρ plots reveals that the TP‐F_4_TCNQ cocrystal features multiple NCIs, including CT interactions, *π–π* interactions, F…H and N…H interactions. These NCIs synergistically stabilize the structure while suppressing intramolecular vibrations, thereby minimizing energy loss in excited‐state molecules and enabling efficient NIR emission. In contrast, Py‐F_4_TCNQ exhibits loosely packed mixed stacking and simpler intermolecular interactions, leading to fluorescence quenching.

In addition, the distance and orientation of intermolecular interactions also affect the optoelectronic properties of the cocrystals. For instance, CT cocrystal (di‐t‐BuPy‐fluoranil) exhibits significant fluorescence quenching and n‐type semiconductor behavior, whereas AP cocrystal (di‐t‐BuPy‐OFN) shows minor fluorescence quenching and insulator properties [77]. These distinct differences arise from different intensities of intermolecular *π–π* stacking. Specifically, the molecular planes in the CT cocrystal are fully parallel, whereas those in the AP cocrystal form a 2.32° angle (Figure 2E). The centroid…centroid distances are 3.271 and 3.471 Å in the CT cocrystal and the AP cocrystal, respectively, indicating slightly stronger CT interaction compared to AP interaction. Furthermore, Hirshfeld surface analysis based on d_norm_ plots reveals that the CT cocrystal surface features multiple small red spots and white regions, signifying stronger intermolecular interactions. In contrast, the AP cocrystal's d_norm_ surface is dominated by white areas and a single weak red spot, suggesting relatively weaker interactions occurring primarily at van der Waals radii. The difference in these *π–π* stacking interactions provides a crucial theoretical insight for regulating the optoelectronic properties of the materials.

**FIGURE 3:**
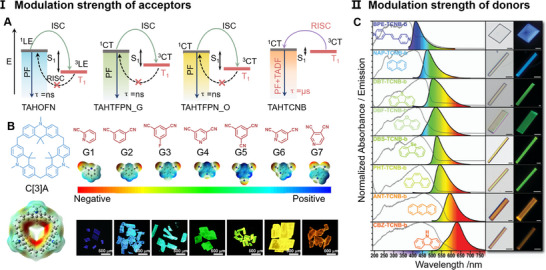
Physicochemical Properties of Cocrystals. (A, B) Modulation energy level of electron acceptors. (A) The effect of the energy level of electron acceptors on achieving thermally activated delayed fluorescence (DATF). Reproduced with permission [60]. Copyright 2023, Springer Nature. (B) The effect of electron cloud density on the emission color of DATF. Reproduced with permission [48]. Copyright 2024, Springer Nature. (C) Modulation energy level of electron donors to achieve different wavelengths of emission (Scale bars: 20 µm). Reproduced with permission [78]. Copyright 2022, The Royal Society of Chemistry and the Chinese Chemical Society.

In CT cocrystals, emission at different wavelengths is achieved by adjusting the strength of the donor/acceptor [67]. For example, selecting a twisted aromatic hydrocarbon (TAH) donor and diverse planar acceptors results in distinct color emissions and TADF [60]. As the acceptor strength increases, the CT degree achieves a transition from LE state to CT state, accompanied by a reduced energy band gap (E_g_) between HOMO and LUMO as well as a decreased splitting energy ΔE_ST_(ΔE_ST_ = E_S1_‐E_T1_). When ΔE_ST_ becomes sufficiently small, reverse intersystem crossing (RISC) occurs, thereby achieving TADF. Besides, RDG and Sign(λ_2_)ρ analyses of TAHTCNB reveal denser weak interactions and significant steric hindrance, which suppress molecular vibrations, minimize energy loss, and further enhance TADF characteristics (Figure 3A). As another example, full‐color‐tunable TADF materials [48] were prepared by precisely modulating the electron attraction ability of the acceptors using the organic macrocyclic compound calix[3]acridan (C[3]A) as the electron donor (Figure 3B). Additionally, regulating the energy levels by varying the type of electron donor [78] can also achieve rainbow emission (Figure 3C).

In addition, by radicalizing and protonating the acceptor, Hu et al. transformed TCNQ into HTCNQ•, which exhibits a higher electron‑accepting ability [13]. This leads to a more pronounced redistribution of electron density between the donor and acceptor, thereby strengthening CT interaction between them. As a result, the originally existing magnetic and conductive properties are significantly enhanced. Besides, Li et al. proposed an anion‐counterion strategy whereby simple ion exchange of counterions (e.g., halide ions) in the donor molecules can strengthen the degree of charge transfer without altering their skeletal structure, thereby enhancing the photothermal conversion efficiency [16].

## 4 Non‐Covalent Synthesis of Hierarchical Structures

Organic cocrystals have emerged as competitive candidates for the construction of organic hierarchical structures (OHSs), benefiting from their high chemical/structural compatibility and tunable optoelectronic characteristics. During the self‐assembly of OHSs, a variety of non‐covalent interactions (NCIs) may be involved due to the different types of cocrystals. Here, we will present the preparation of OHSs in two parts: self‐assembly driven by single interaction and multiple interactions.

### 4.1 Self‐Assembly Driven by Single Interaction

**FIGURE 4:**
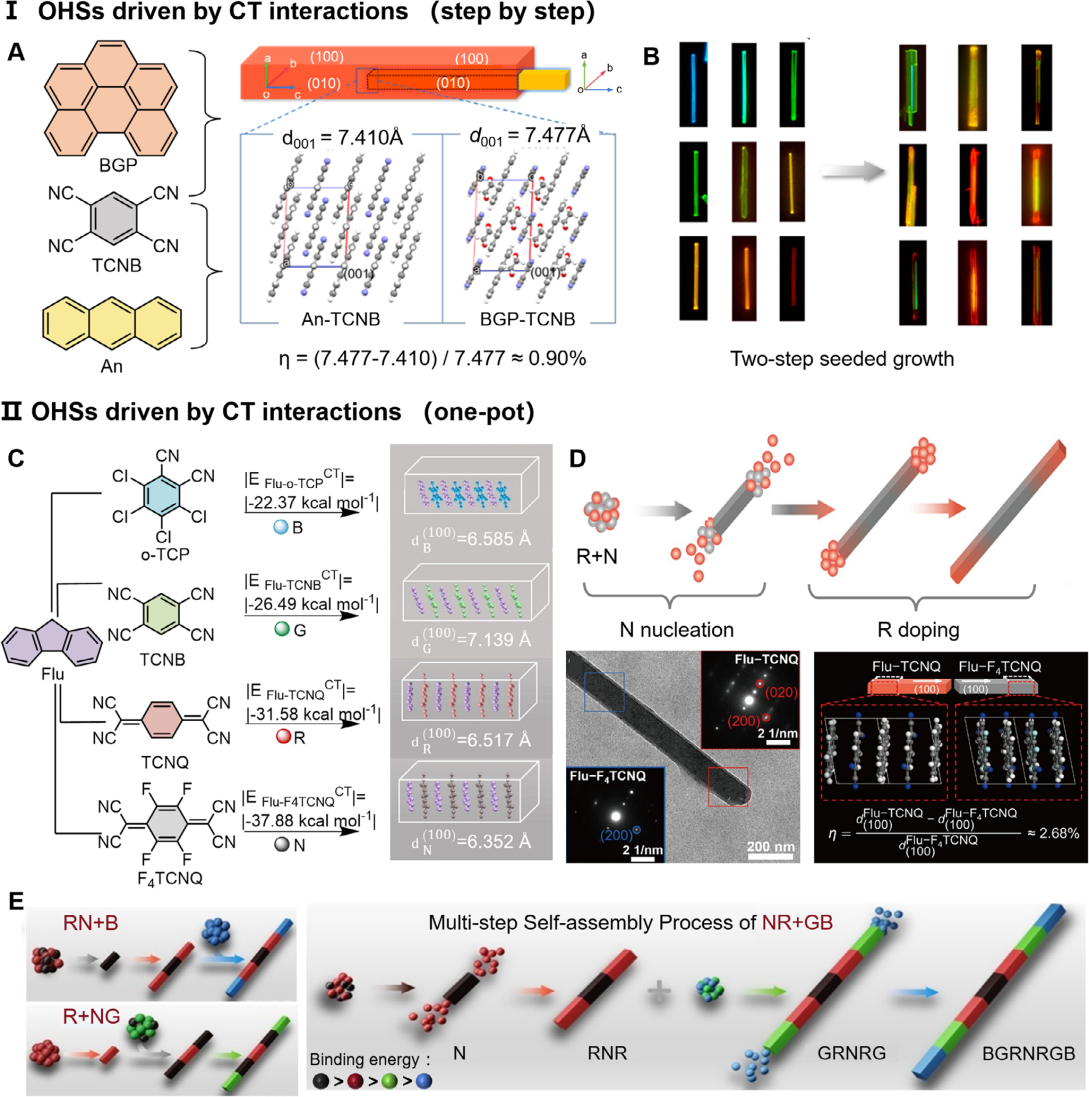
Self‐assembly driven via a single interaction (CT interaction). (A, B) Nucleation order step by step. (A) Core/shell structures and lattice matching. Reproduced with permission [84]. Copyright 2020, American Chemical Society. (B) The library of core/shells. Reproduced with permission [84]. Copyright 2020, American Chemical Society. (C–E) Nucleation order by the one‐pot method. (C) Four types of Flu‐based cocrystals on the strength of CT interaction and similar packing on the (100) plane. Reproduced with permission [79]. Copyright 2024 Wiley‐VCH GmbH. (D) Schematic diagram of sequentially nucleating to form a gradient block, with the TEM image. Inset: SAED patterns recorded at the middle & tip for blocks, and lattice match. Reproduced with permission [86]. Copyright 2024, Science China Press. (E) Universality of programmable self‐assembly. Reproduced with permission [79]. Copyright 2024 Wiley‐VCH GmbH.

Common fabrication strategies for OHSs include one‐pot method [79, 80], seeded growth [29, 46], crystal welding [81, 82], mechanical stripping and transfer [83], and so on, though this section focuses on the first two approaches. In seeded growth, initially dropped precursor solutions preferentially nucleate to form crystalline seeds, followed by sequential nucleation of later introduced solutions. Crucially, the selected molecules should have good chemical and structural compatibility to achieve a better lattice match. Fu et al. developed a library of core/shell configurations through a two‐step solution crystallization strategy [84], using shorter wavelength CT cocrystals as seeds and longer wavelength‐emitting CT cocrystals as the shell layer (Figure 4B). Specifically, benzo[ghi]perylene (BGP)‐TCNB solution is added dropwise to crystallize first as a crystal seed, and then Anthracene (An)‐TCNB is added dropwise to nucleate on the surface of BGP‐TCNB and grow laterally epitaxially to form a core/shell structure. In addition, BGP‐TCNB and An‐TCNB have similar molecular arrangements (Figure 4A), suggesting that they are well compatible. The lattice mismatch (η = |d_1_‐d_2_|/d_1_, where d_1_ and d_2_ are the lattice spacings of the substrate and secondary building block, respectively) is calculated to be 0.9% on the (100) plane, leading to epitaxial growth of the shell layer BGP‐TCNB shell along [99] direction. The core/shell structure requires excessive lattice matching, and if the lattice mismatch is large (>3%), a block or branch structure may be generated. Notably, reversed core/shell [85] or block [86] can be fabricated by exchanging the dropping order of precursor solutions using the stepwise seed growth approach.

Another method, the one‐pot method, involves mixing all components homogeneously in one system, and according to the strength of the interactions between D and A, the strongly interacting cocrystals preferentially nucleate, then the weakly nucleate sequentially. For example, our group synthesized NRN block structures [86] with gradual doping and a gradient of concentration distribution, utilizing Flu‐TCNQ cocrystal (R) and Flu‐F4TCNQ cocrystal (N), as shown in Figure 4D. Impressively, building upon two primary cocrystals, we achieved ordered nucleation of N, R, G, B architectures, choosing Flu‐TCNB(G) and Flu‐o‐TClP(B), based on CT interaction strengths: |−37.88 kJ/mol| > |−31.58 kJ/mol| > |−26.49 kJ/mol| > |−22.37 kJ/mol|. Furthermore, these cocrystals on (100) exhibit a similar 1D face‐to‐face stacking pattern and face spacing (Figure 4C), which contributes to the nice lattice matching between different blocks. Based on the above two key factors, the BGRNRGB seven‐block [79] (Figure 4E) was successfully fabricated.

In summary, seeded growth is an artificial control of the nucleation sequence through the order of precursor solution addition, while the one‐pot method is a spontaneous co‐assembly triggered by the strength of NCIs. The former has a cumbersome process and difficult control of the crystal growth, but the presence of high‐quality seeded crystals provides a template for subsequent material growth, leading to OHSs with better crystallinity and fewer defects. Though the latter process is simple, the complexity of the system leads to a variety of structures, and the preparation of complex structures is more difficult. The introduction of NCIs is conducive to the realization of controllable and orderly growth of building blocks in OHSs, which provides a feasible pathway for the construction of accurately constructed complex OHSs.

### 4.2 Self‐Assembly Driven by Multiple Interactions

**FIGURE 5:**
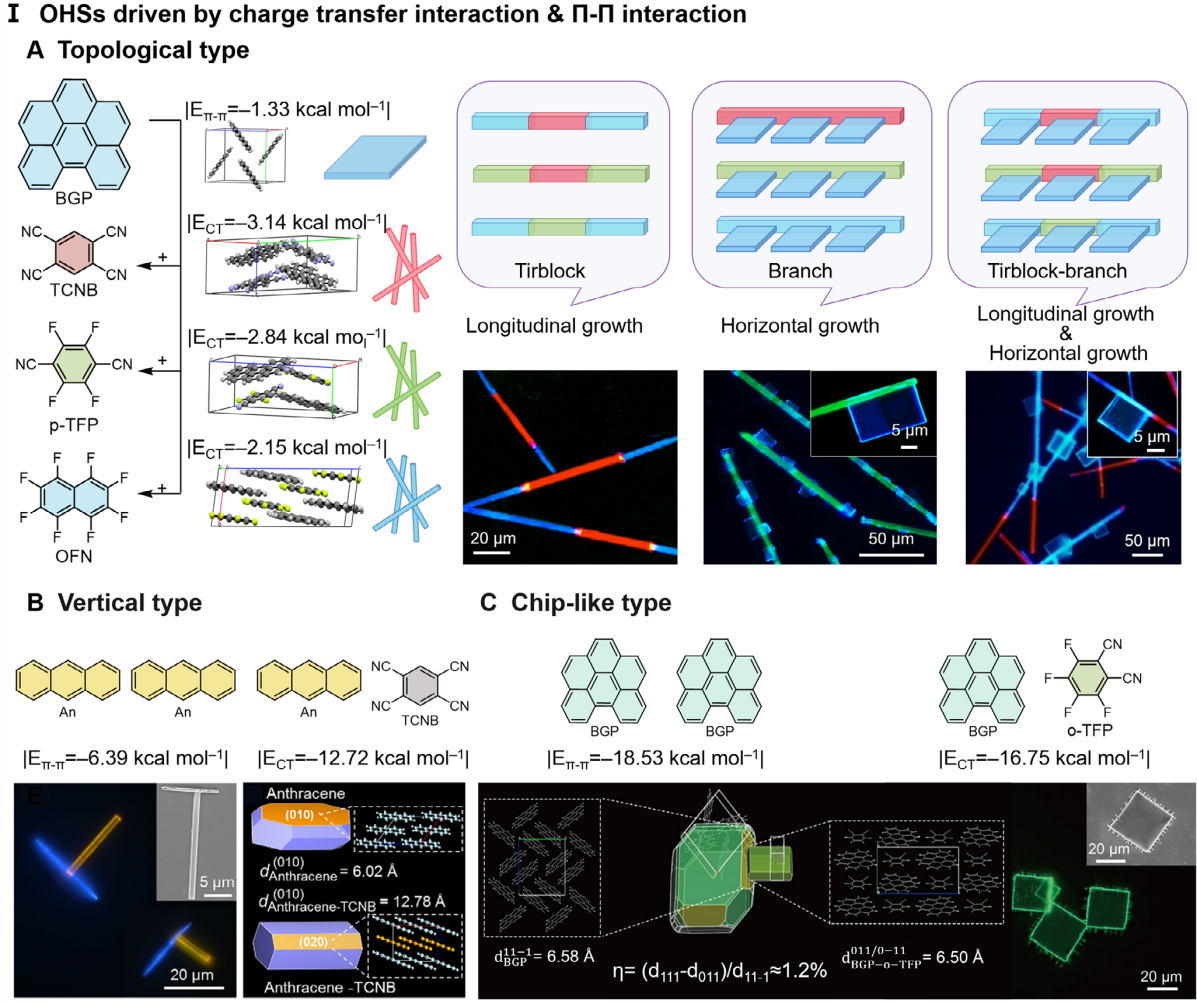
Self‐assembly driven via Multi‐interaction based on lattice match (CT interaction & *π–π* interaction). (A) Sequential self‐assembly of topological structures. Reproduced with permission [87]. Copyright 2020, Chinese Chemical Society. (B) Self‐assembly of vertically growing branches. Reproduced with permission [88]. Copyright 2020, American Chemical Society (C) Self‐assembly of chip‐like heterostructures. Reproduced with permission [89]. Copyright 2020, Science China Press and Springer‐Verlag GmbH Germany.

Compared with the hierarchical self‐assembly driven by a single NCI, OHSs formed by multiple NCIs exhibit more complex and diverse structures, and the hierarchical structures prepared by utilizing the synergistic effect of multiple interactions usually have higher stability. For example, the selective introduction of CT interactions and *π–π* interactions enables the precise construction of a series of topological OHSs [87]. The transformation from BGP to BGP‐based cocrystals was realized by regulating intermolecular NCIs, further constructing triblock structures, branches, and triblock branches (Figure 5A). BGP molecules self‐assemble into 2D microsheets via *π–π* interactions, while BGP with TCNB, p‐tetrafluoroterephthalonitrile (p‐TFP), and octafluoronaphthalene (OFN) self‐assembles into 1D microrods via CT interactions. Using the sequential growth in a one‐pot method, BGP‐TCNB was referentially nucleated. Based on the lattice‐matching principle, BGP‐OFN grew longitudinally to form a triblock. Furthermore, by adjusting the stoichiometric ratio of BGP to TCNB, horizontal growth of BGP microsheets on the surface of BGP‐TCNB microrods was achieved, constructing branch‐like structures. By combining the above two self‐assemblies, the BGP will grow laterally along the surface of the triblock, thus constructing a triblock‐branch. This developed a generalized strategy for the precise construction of organic topological heterostructures by changing the type of electron acceptor. Additionally, the vertically grown branching structure was precisely constructed by rationally regulating the *π–π* interactions between anthracene (An) molecules and the CT interactions between anthracene (An) and TCNB [88] (Figure 5B). The An‐TCNB cocrystal (|E_CT_ = |−12.72 kcal/mol|) preferentially formed the yellow microrods, followed by the blue An single crystals (|Eπ‐π = |−6.39 kcal/mol|) grows vertically at the end of the rods. Both crystals adopt a similar face‐to‐face stacking mode, with a lattice matching rate of 94.2% on the (010) plane. Similarly, chip‐like heterostructures were prepared according to the above strategy [89] (Figure 5C). Notably, during the preferential growth process of BGP molecules, a micro‐etching process occurred on the surface, releasing BGP molecules. The released BGP combined with o‐TFP to form cocrystal microrods, while the etching provided nucleation sites for the cocrystals. The lattice mismatch rate between the two crystals was only 1.2%.

**FIGURE 6:**
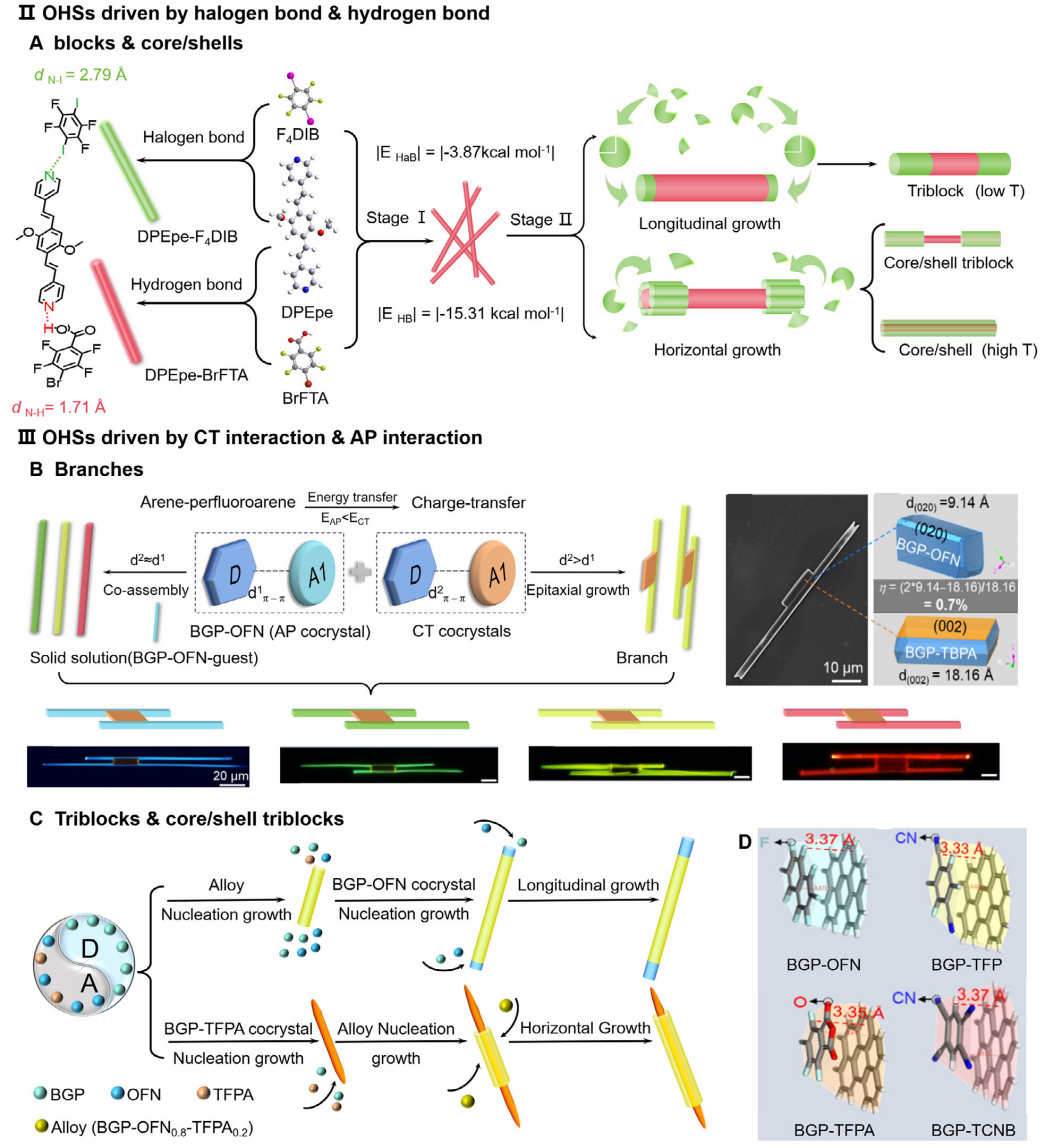
Self‐assembly Driven via Multi‐interaction. (A) Blocks & core/shells driven via HB & HaB. Reproduced with permission [90]. Copyright 2019, Springer Nature. (B, C) OHSs driven via CT & AP interactions. (B) Branches driven via CT & AP interactions. Reproduced with permission [91]. Copyright 2023, Chinese Chemical Society. (C) Triblocks & core/shell triblocks driven via CT & AP interactions. Reproduced with permission [92]. Copyright 2022, Springer Nature. (D) The intermolecular distances of BGP‐based cocrystals. Reproduced with permission [91]. Copyright 2023, Chinese Chemical Society.

Hydrogen bond (HB) and halogen bond (HaB) possess directionality and saturability, playing vital roles in molecular self‐assembly and material stability. Due to the stronger HB between DPEpe and 4‐bromo‐2,3,5,6‐tetrafluorobenzoic acid (BrFTA), the two preferentially self‐assembled into red‐emissive DPEpe‐BrFTA seeded nanowires; 4,4'‐((1E,1'E)‐(2,5‐dimethoxy‐1,4‐phenylene) bis(ethene‐2,1‐diyl)) dipyridine (DPEpe) and 1,4‐diiodotetrafluorobenzene (F_4_DIB) self‐assembled into green‐emissive cocrystal nanowires via HaB [90] (Figure 6A). Epitaxial growth of DPEpe‐F_4_DIB on the tips or lateral of DPEpe‐BrFTA cocrystals was achieved by controlling the solution temperature and the ratio of HB/HaB cocrystals to form triblocks or core/shell nanowires. Lattice matching and surface interface energy balance promote the formation of triblock and core/shell structures, respectively.

Last but not least, CT interaction and AP interaction promote the self‐assembly of OHS through a dynamic competitive and synergistic balance. For instance, the low energy bandgap of CT cocrystals enables them to accept excitation energy from BGP‐OFN (an AP cocrystal) [91]. This energy transfer process from AP to CT cocrystals achieves full‐color emission and enhances the overall luminescence efficiency of the system. Moreover, by tuning the intermolecular distance (d_π–π_ from 3.33 to 3.48 Å) (Figure 6D), organic solid solutions and organic lateral hierarchical structures featuring tunable structural configurations and optical properties were selectively constructed. The similar intermolecular distances and stacking patterns reflect the excellent structural compatibility of the components (The lattice mismatch rate of BGP‐OFN@BGP‐TBPA single‐lateral branch was only 0.3%). Systematic tuning of compositional proportions enabled the fabrication of single lateral and bilateral branched nanostructures (Figure 6B). Further, still in the same molecular system, lattice‐mismatch‐free growth of OHSs was prepared, and a large number of them were significantly prepared. The similarity of molecular size and stacking distance between the cocrystal molecules facilitates homogeneous nucleation to form the organic alloy BGP‐OFN_0.8_‐TFPA_0.2_, which reduces phase separation [92]. Finally, the sequence of formation (organic cocrystal or organic alloy) was determined by adjusting the component ratios and the type of poor solvent to control the solubility of BGP‐TFPA, ultimately leading to the construction of triblock nanowires or core/shell triblock nanowires (Figure 6C).

In summary, hierarchical self‐assembly driven by single NCIs typically yields relatively simple and regular structures. In contrast, self‐assembly driven by multiple NCIs, the complexity of the strength and direction makes it more difficult to achieve precisely regulated self‐assembly, but it is more advantageous in constructing multifunctional hierarchical structures.

The examples mentioned in the above figure are all sequential self‐assembly in solution systems. Additionally, through stepwise physical vapor deposition (PVD), 2D OHSs were prepared in large‐scale based on CT cocrystals [93]. Benzo[b]benzene‐[4,5]thieno[2,3‐d]thiophene (BBTT) and TCNB (BTB) form 1D yellow rod‐like structures in liquid‐phase systems. However, the morphology can be transformed from 1D into 2D hexagons utilizing the PVD method. Subsequently, serving the yellow BTB as templates, the vertical growth of blue Na‐TCNB (NTB) on the hexagonal substrate can be induced. Further adjustment of the deposition time for the two cocrystals enables the fabrication of vertically structured OHSs with white‐light emission.

Furthermore, stepwise sequential self‐assembly is also applicable to 3D OHSs [94]. This is exemplified by 6,12‐dihydroindeno[1,2‐b]fluorene (IDF) and TCNB, which form cubic DFNB cocrystals via CT interaction‐driven, 3D epitaxial growth. By comparing the attachment energies on different crystal facets, the growth sequence of the shell component—the Dibenzo[d,d′]benzo[1,2‐b:4,5‐b′]dithiophene (IDT)‐TCNB cocrystal (DTNB)—on the various faces of the cubic DTNB cocrystals can be inferred. Higher attach energy (with greater absolute value) corresponds to stronger binding and prioritized growth. Following the (011), (01–1), and (100) growth order, DTNB ultimately forms a complete core–shell structure. These examples convincingly demonstrate the broad applicability and generality of the NCIs‐driven “sequential self‐assembly” strategy.

Notably, OHSs prepared via solution‐based methods are highly susceptible to environmental influences, especially for systems involving the simultaneous construction of metastable and stable structures. Factors such as ambient temperature, humidity, and solute concentration gradients can all significantly impact the final OHSs. Therefore, during the solution‐based preparation of OHSs, the resulting structures may not only contain more undesired configurations but also some serendipitously favorable ones. Additionally, it is not uncommon for experimental results to be challenging to reproduce precisely. In contrast, the preparation of PVD offers much better controllability, yielding crystals that are purer, more uniform in size, and contain fewer defects. However, OHSs produced by PVD are generally limited to smaller sizes, and some unique structural features achievable through solution‐based methods may not be attainable via PVD. Therefore, the choice of the most suitable method should be based on a comprehensive consideration of the respective advantages and limitations in practice.

### 4.3 Learning for Prediction and Screening

In addition to the aforementioned method of utilizing the binding energy of cocrystals for the targeted construction of OHSs, machine learning approaches can also be employed to screen the desired cocrystal candidates [95], which further provide guidance for constructing OHSs efficiently and precisely.

Ji et al. proposed a machine learning (ML) framework driven by both a “drug—conformer—solvent” ternary database and thermodynamic mechanisms. This method not only can predict suitable coformers but also can screen appropriate solvents, addressing the limitation of traditional methods that focus solely on coformers while neglecting the influence of solvent effects (Figure 8A) [96]. First, a systematic comparison of seven mainstream machine learning algorithms reveals that Extreme Gradient Boosting (XGB) demonstrated the best overall performance in predicting cocrystal formation (Figure 8B). To further optimize feature inputs, 20 key features are selected from nearly 400 molecular descriptors. The corresponding heatmap of low correlations confirms the representativeness and non‐redundancy of the feature set, ensuring the model's robustness and interpretability (Figure 8C). Parameters are integrated into the XGB model from three thermodynamic models (HSP: Hansen Solubility Parameters, COSMO‐RS: Conductor‐like Screening Model for Real Solvents, and PC‐SAFT: Perturbed‐Chain Statistical Associating Fluid Theory). Then the CREATE model (the predictive model for co‐crystal Coformer and Reaction solvent screening based on Extreme gradient boosting algorithm Assisted by multiple Thermodynamic mechanisms) predictive framework is constructed. Performance comparisons indicate that integrating multi‐mechanism features significantly enhances the model's discriminative capability. Among these, CREATE achieved the highest accuracy and F1 score, robustly demonstrating the synergistic advantages of combining physical mechanisms with data‐driven methods (Figure 8D). In summary, this work achieved efficient and highly accurate prediction of cocrystal formation through an integrated strategy of algorithm optimization, feature engineering, and mechanism integration.

Another example, Jiang et al. developed a data‐driven approach that integrates machine learning with high‐throughput automation to accelerate the discovery of polar organic cocrystals [97]. Based on the screening of cocrystal candidates, polar cocrystals were further identified through model prediction and experimentally validated via an automated workflow.

**FIGURE 7:**
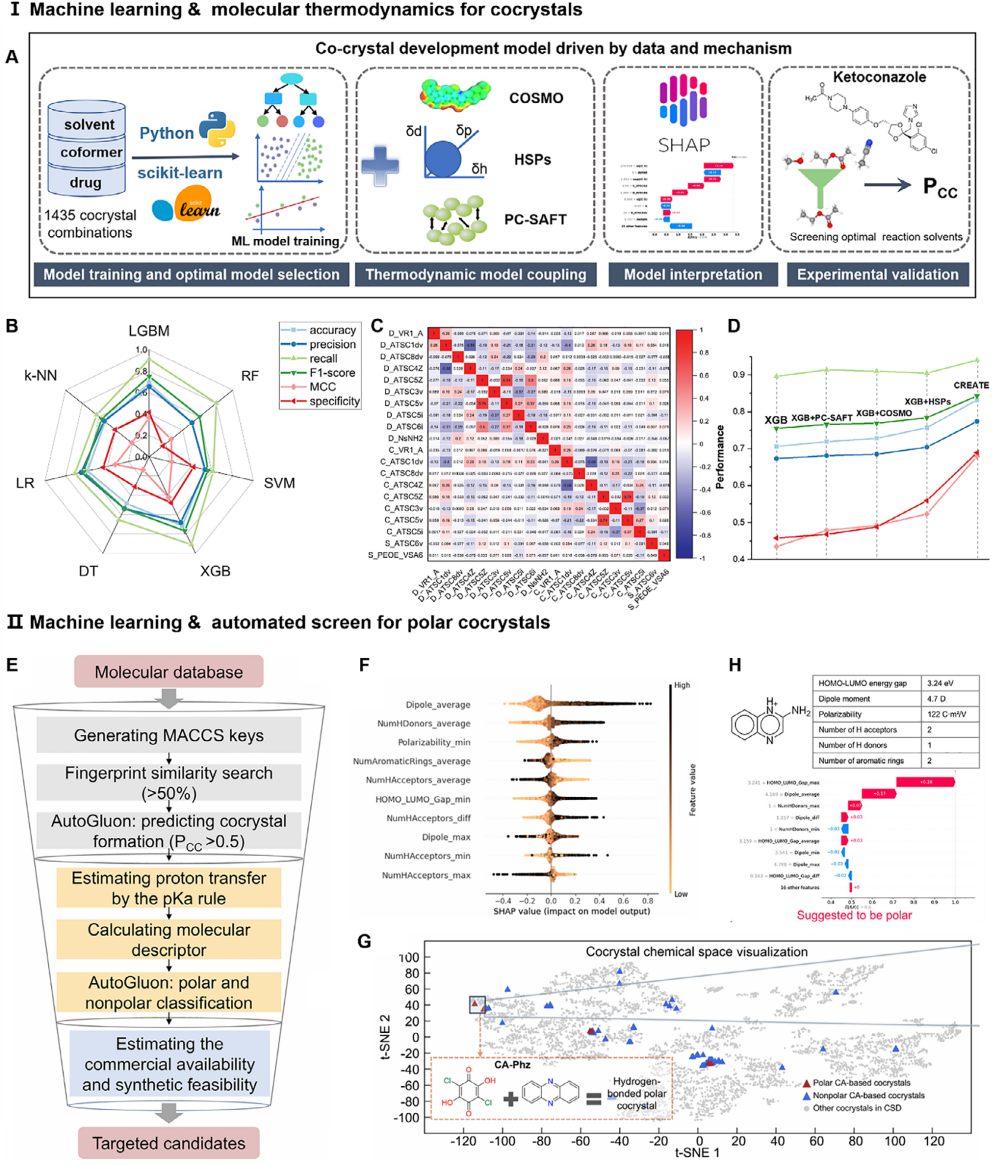
Machine learning for cocrystals. (A) The workflow of predict conformer and reactions based on machine learning and thermodynamic modeling. (B) The radar chart of various predictive performances based on different machine learning methods. (C) Heatmap of correlations between the selected molecular descriptors (20‐input features). (D) The predictive performances integrated machine learning and multiple thermodynamic models. (A–D), reproduced with permission [96]. Copyright 2025, Wiley‐VCH GmbH. (E) Flow chart of the screening process. (F) Shapley Additive Explanations (SHAP) beeswarm plot illustrating the impact of features on polar cocrystal classification. (G) Polar/nonpolar cocrystal chemical space visualization by t‐distributed stochastic neighbor embedding (t‐SNE). (H) Waterfall plots illustrate model predictions for a polar cocrystal, showing its molecular structure and calculated feature values in a waterfall plot. (E–H) reproduced with permission [97]. Copyright 2025, American Chemical Society.

Chloranilic acid (CA) was selected as the cocrystal former due to its ability to act as both a proton donor and acceptor, facilitating the formation of hydrogen‐bonded networks. Before predicting polar cocrystals, it is first necessary to determine whether a molecular pair can form a cocrystal, followed by polarity prediction. To predict whether a pair of molecules could form a cocrystal, the model does not directly process the 3D structures of the two molecules. Instead, the MACCS (Molecular ACCess System) fingerprints of the two molecules are concatenated or combined to form a longer feature vector representing the “molecular pair.” The MACCS fingerprint is a classical molecular structural fingerprint in cheminformatics that encodes key internal molecular information, such as “whether a benzene ring is present” or “whether heteroatoms are present.” If a certain structural feature is present, it outputs 1; otherwise, it outputs 0. By compiling answers to many such questions, a unique binary barcode is generated for each molecule. MACCS enables a rough screening of molecules in the database by comparing the “presence or absence” of functional groups. The machine learning model (AutoGluon) is then used to predict the likelihood of forming a cocrystal with CA, with a probability threshold of Pcc > 0.5 serving as a strict screening criterion (Figure 7E).

In the second stage, more precise DFT‐derived descriptors are used for polarity prediction of the screened cocrystal candidates. Factors influencing cocrystal polarity, such as the HOMO‐LUMO energy gap and dipole moment, are quantified using SHapley Additive exPlanations (SHAP), as shown in Figure 7F. Positive SHAP values represent favorable contributions, with larger values indicating greater importance, while negative values indicate unfavorable contributions. A machine learning dimensionality reduction algorithm, t‐SNE, is employed to visualize the data after polarity screening in two dimensions (Figure 7G). Red (polar) and blue (nonpolar) triangular points are intermingled without forming completely separated clusters. This visually reinforces the difficulty of absolutely predicting crystallization polarity solely from molecular components. Therefore, more detailed descriptors (HOMO‐LUMO energy gap, dipole moment, etc.) should be introduced to narrow the scope of screening. Furthermore, inputting the calculated descriptors into AutoGluon ultimately outputs a binary classification result to determine the polar/nonpolar nature of the cocrystals. (Figure 7H).

In summary, this strategy offers a novel path to construct OHSs utilizing machine learning to screen cocrystals broadly. Building upon this foundation, we could obtain suitable cocrystal candidates first and then proceed to construct OHSs, which enhances the accuracy and efficiency of the self‐assembly process.

## 5 Application‐Oriented Self‐Assembly

Traditional self‐assembly research typically starts from the fundamental properties of molecules to explore their naturally formed structures and properties. In contrast, application‐oriented self‐assembly is based on the reverse design of molecular structures and assembly processes to realize specific functions from the needs of target applications. For example, the strength of intermolecular interactions is modulated by introducing specific functional groups to realize the fluorescence emission of the organic cocrystal at different wavelengths. And then the designed organic cocrystal units are self‐assembled into functional integrated hierarchical structures by NCIs. Throughout this process, targeted optimization of the crystals is achieved by adjusting ambient conditions (e.g., solvent type /temperature/ component ratios), ultimately constructing materials with tailored functionalities. The following sections will introduce this approach from three perspectives: light generation, light propagation, and light detection.

### 5.1 Solid‐State Lasers

**FIGURE 8:**
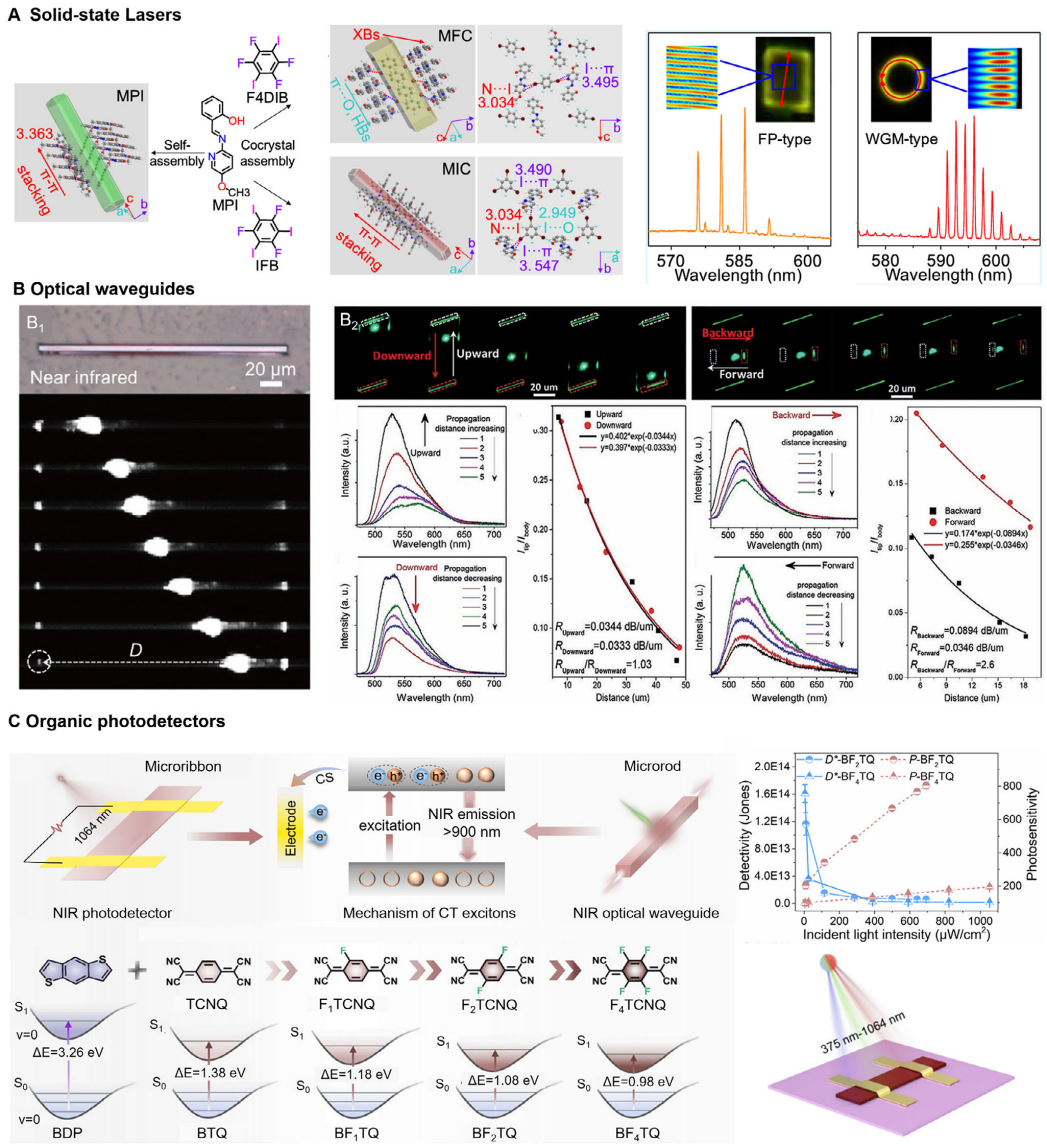
Application‐oriented self‐assembly of cocrystals. (A) Tailoring the cavity structures of organic cocrystal microlasers. Reproduced with permission [98]. Copyright 2019, American Chemical Society. (B) Optical waveguides. (B_1_) 1D waveguides of NIR cocrystals. Reproduced with permission [50]. Copyright 2024, Science China Press. (B_2_) 2D waveguides. Reproduced with permission [35]. Copyright 2018, Wiley‐VCH Verlag GmbH & Co. KGaA, Weinheim. (C) Organic photodetectors. Reproduced with permission [105]. Copyright 2024, American Chemical Society.

In the context of organic solid‐state lasers, fluorescence quenching frequently occurs in aggregated organic molecules due to intermolecular *π–π* stacking and dipole‐dipole interactions, reducing emission efficiency. As exemplified by 1,4‐bis(2‐cyanostyryl) benzene (o‐BCB), packs with short intermolecular distances promote excimer formation. The introduction of the HaB donor 4‐bromo‐2,3,5,6‐tetrafluorobenzoic acid (BFC) through a cocrystal engineering resulted in increased intermolecular distances (from 3.06 to 7.10 Å), shortened fluorescence lifetime (from 11.6 to 0.8 ns), while enhancing radiative transition rate, which realized a transition from non‐lasing to lasing transition [98]. Furthermore, to achieve more efficient population inversion, distinct components can be selected to tailor energy levels and cavity structures [99]. The four‐level proton‐transfer compound (E)‐2‐(((5‐Methoxypyridin‐2‐yl)imino)methyl)phenol (MPI) was chosen as the HaB acceptor, while 1,4‐diiodotetrafluorobenzene (F_4_DIB) and 1,3,5‐trifluoro‐2,4,6‐triiodobenzene (IFB) served as HaB donors, forming organic cocrystal MFC and MIC, respectively. MFC forms a 2D sheet‐like structure (Fabry‐Perot (FP) type) with pronounced optical waveguide properties and strong 1D field confinement; MIC forms a cyclic structure (Whispering Gallery Mode (WGM) type) with total reflection characteristics, rendering them suitable for optical resonance (Figure 8A). The introduction of halogen bonds not only preserves the original energy level structure but also enhances the degree of intermolecular charge transfer and promotes the reduction of excited state energy, which realizes the tuning of laser performance.

Significantly, the difficulty in realizing stimulated emission within organic molecular crystals lies in the molecular stacking arrangement. Cocrystallization strategies that modulate intermolecular interactions and alignments can enhance stimulated emission performance. Tian et al. employed an HB cocrystallization approach: cocrystals CHQ and CFHQ formed J‐aggregate and herringbone aggregate structures, respectively, reducing *π–π* stacking and shortening excited‐state lifetimes [8]. This enabled low‐threshold stimulated emission with high gain characteristics. This enabled low‐threshold stimulated emission with high gain characteristics. In addition, Geetha Bolla et al. modulated the stacking structure of the molecule by changing the type of HaB cocrystal (from C–Br···N to C–I···N) [100]. From DPYA‐BrFB (mixed stacking) to DPYA‐IFB (isolated stacking) is achieved by reducing the low‐frequency vibrational modes and enhancing the intermolecular interactions to achieve lower amplified spontaneous emission (ASE) thresholds and higher radiative decay rates. Notably, macrocyclic compounds leverage their cavity structures to precisely control host‐guest interactions for excellent lasing performance. Pillar[5]arene (P5) acts as a host that precisely encapsulates the guest molecule trans‐4‐[p‐(dimethylamino)styryl]‐1‐methylpyridinium iodide (DASP), forming stable nanocapsule structures [101]. This spatial confinement effectively suppresses the twist intramolecular charge transfer process and aggregation‐caused quenching (ACQ) in DASP molecules, thus enabling highly efficient light emission and low‐threshold laser output. Thus, cocrystallization paves the way for designing high‐performance organic laser materials with broad applicability in photonics.

### 5.2 Optical Waveguides

Organic cocrystals can not only achieve laser generation but also enable efficient optical waveguides, which are crucial components in many integrated micro/nanophotonic devices. Due to their smooth surfaces and uniform internal structures, organic cocrystal single crystals can achieve extremely low optical transmission loss. By tuning the bandgap between the HOMO and LUMO of CT cocrystals, 1D cocrystals emitting from the visible to near‐infrared range can be prepared. According to the relation *I_tip_/I_body_ = A exp(‐RD)*, where *I_tip_* represents the emission intensity at the crystal tip, I_body_ represents the emission intensity at the excitation spot, and *R* represents the optical loss efficiency [102]. As the excitation site moves, the distance between the input and output ends gradually increases, and the PL intensity gradually decreases (Figure 8B_1_) [50].

Compared with 1D cocrystals, 2D cocrystals allow photon transmission along different directions, making them more suitable for chip‐scale photonic devices. As shown in Figure 8B_2_, the 2D optical waveguide based on a cocrystal of 4,4’‐((1E,1’E)‐(2,5‐dimethoxy‐1,4‐phenylene)bis(ethene‐2,1‐diyl))dipyridine (DPEpe) and F_4_DIB exhibits symmetric optical waveguide (with similar optical loss coefficients) along the (100) direction as well as asymmetric waveguide (with different coefficients along the two opposite directions) along (010) direction, further demonstrating the potential of cocrystals in miniaturized optoelectronic devices [35]. Unconventional morphologies such as branched cocrystals [103] and parallel grouping cocrystals [104] provide additional practical support for realizing complex photon transmission pathways.

### 5.3 Organic Photodetectors

Above, light generation and transmission are designed through a series of processes utilizing the self‐assembly of organic cocrystals. Furthermore, organic cocrystals have been directionally engineered to achieve photodetection. Fundamentally, organic cocrystal‐based photodetection involves three key processes: the formation of CT excitons, charge separation, and photocurrent generation. (i) Generation of CT excitons: Under photoexcitation, electrons transition from the HOMO of the D to the LUMO of the A, forming electron‐hole pairs (excitons). (ii) Charge separation of CT excitons: Under an applied electric field, CT excitons undergo charge separation, causing electrons to migrate toward the anode and holes toward the cathode, establishing directional charge transport. (iii) Photocurrent generation: The separated free carriers are collected by the electrodes, generating photocurrent. Compared to the dark current, the photocurrent is significantly enhanced, enabling efficient photodetection. In general, it is a process of converting light signals into electrical signals.

In our group, the E_g_ of organic cocrystals was modulated by increasing the number of F in electron acceptors [105]. This enabled high‐efficiency photodetection in the NIR‐II region (D^*^ > 10^1^ ^4^ Jones) and low‐loss optical waveguiding (α < 0.01 dB/µm) (Figure 8C). In addition, the interpenetrating network formed by the donor and acceptor in the CT cocrystal structure is conducive to efficient charge carrier transport. Additionally, the rigid molecular structures within the cocrystal can effectively suppress non‐radiative transitions caused by molecular vibrations, thereby enhancing the charge separation process. Hu et al. demonstrated that large‐area 2D cocrystal films exhibit device performance enhanced by over 100 times compared to devices based on micro‐cocrystals of the same components [106]. Besides, selecting asymmetric electrodes effectively suppresses dark current, leading to improved carrier collection efficiency. Moreover, Huang et al. developed a wide‐spectrum (365–1050 nm), fast‐responding (150 ms), retina‐like visual sensor with bio‐synaptic functionality using organic cocrystals, offering a novel pathway for biomimetic vision systems [107].

By constructing complex hierarchical architectures utilizing cocrystals, we aim to integrate functionalities like lasing, optical waveguiding, and photodetection into a single structure. This integrated approach offers an element with multifunctional optoelectronic characteristics, paving the way for miniaturized organic photonic circuits.

### 5.4 Multiwavelength

**FIGURE 9:**
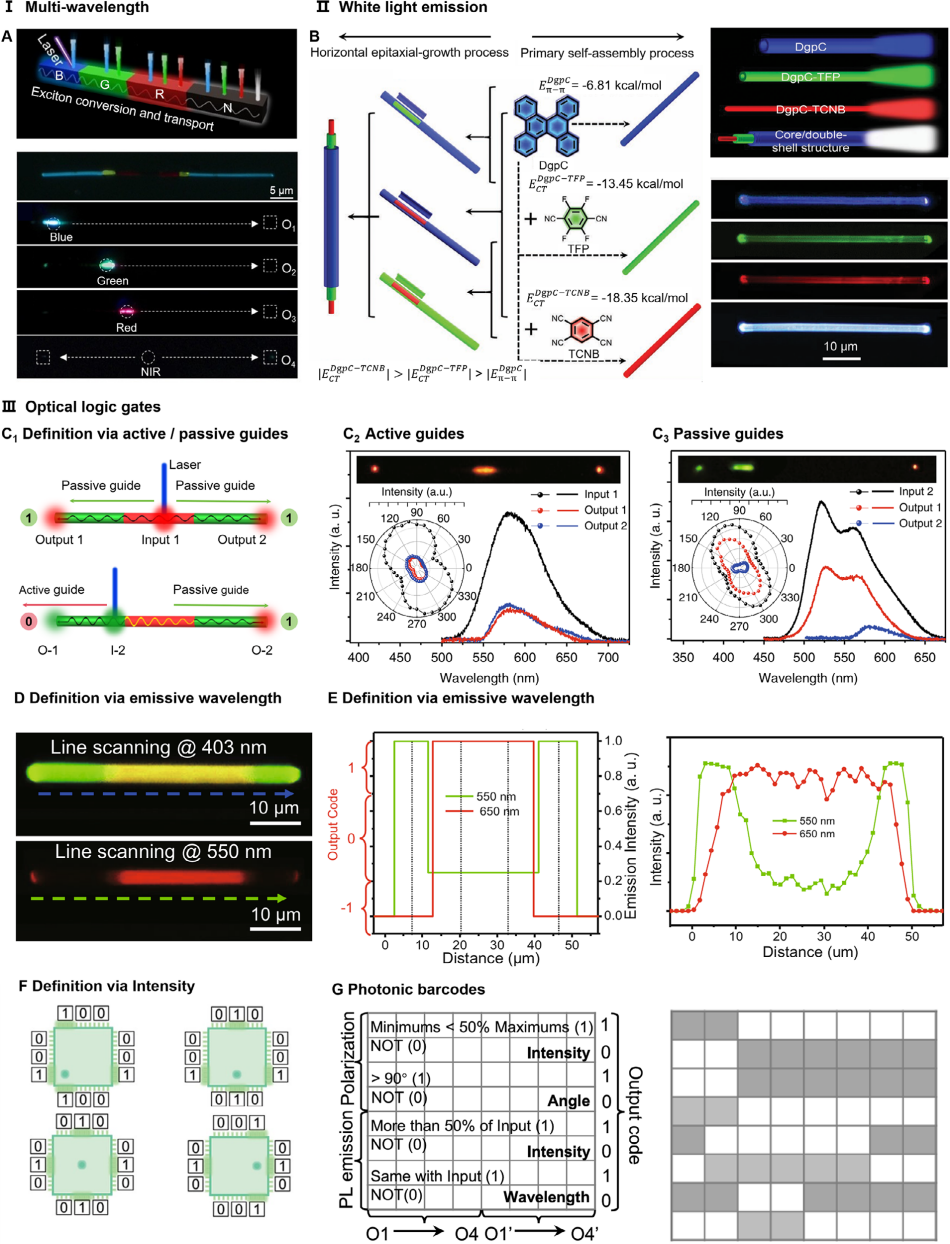
Application‐oriented self‐assembly. (A) Multicolor emission via exciton conversion and transport for multi‐blocks. Reproduced with permission [79]. Copyright 2024, Wiley‐VCH GmbH. (B) Trichromatic white light sources by core/multi‐shells. Reproduced with permission [20]. Copyright 2021, Wiley‐VCH GmbH. (C–F) Optical logic gates (OLGs). (C) OLGs are defined via active/passive guides. Reproduced with permission [90]. Copyright 2019, Springer Nature. (D) OLGs are defined via the emissive wavelength. Reproduced with permission [85]. Copyright 2021, Springer Nature. (E) OLGs are defined spatially via emission intensity. Reproduced with permission [85]. Copyright 2021, Springer Nature. (F) Photonic barcodes. Reproduced with permission [46]. Copyright 2024, Springer Nature.

Optical waveguide applications of organic cocrystals from 1D to 2D can be realized by precisely tuning NCIs. Building on this foundation, further control of NCIs and self‐assembly conditions facilitates the construction of hierarchically assembled organic structures with multi‐level assembly characteristics. When building blocks with wide E_g_ are excited, the generated light couples to the building blocks with narrow E_g_ via Förster resonance energy transfer (FRET), yielding dual‐wavelength emission. Following this strategy, four fluorene‐based cocrystals were selected to construct a seven‐segment BGRNRGB structure through multi‐step in situ self‐assembly [79]. Upon exciting the B segment, excitons transfer sequentially: B→G→R→N (Figure 9A). This cascaded energy conversion facilitates efficient transfer and conversion of optical signals across segments, achieving multi‐wavelength output.

### 5.5 White Light Emission

The aforementioned strategy of adjusting dopant ratios not only enables multicolor emission but also facilitates the modulation of white light (WL) sources. For instance, precise control over the doping ratio of guest Py‐TCNB in Py‐OFN (0.5%, 1%, 3%) achieved emission spanning blue, white, and yellow [108]. The generation of WL involves an energy transfer from Py‐OFN to Py‐TCNB, which is based on a similar stacking pattern and the distance between neighboring molecules. Beyond complementary color mixing, WL emission can also be obtained by combining RGB trichromatic cocrystal [109, 110]. Furthermore, WL emission can be realized in heterostructures constructed via hierarchical self‐assembly of three primary color crystals [20]. Based on this principle, three crystals were selected: blue‐emitting dibenzo[g,p]chrysene (DgpC), green‐emitting DgpC‐TFP cocrystal, and red‐emitting DgpC‐TCNB cocrystal (Figure 9B). Through rationally modulating π–π interactions and CT interactions, radially ordered self‐assembly of the trichromatic layers was achieved. The synergistic interplay within the core/double‐shell microwire promotes white‐light generation. More impressively, this self‐assembly approach, driven by elaborate manipulation of NCIs, is universally applicable. By adjusting the stoichiometric ratios of components, the shell thickness and emissive color of the core/shell microwires can be precisely controlled. This tunability enables customization of the microwires for diverse applications, offering a versatile platform for advancing integrated photonics.

### 5.6 Optical Logic Gates

In addition, in organic heterostructures, optical logic operations can be realized through energy transfer between different components and optical waveguide processes [111]. During the process, the intensity/wavelength/polarization of the output signal of the photon may be different from the input signal to define different logic states. In defining optical logic gates, the concepts of active guide and passive guide are often mentioned. Active waveguides primarily facilitate light generation, amplification, and active signal processing, whereas passive waveguides transmit optical signals without energy conversion. When photons propagate through the same media exhibiting identical emission characteristics at excitation and output sites, this constitutes “active waveguiding.” For heterogeneous systems, waveguide classification depends on spectral/polarization consistency between input and output: consistent signatures indicate passive waveguiding (Figure 9C_2_), while inconsistencies are active waveguides (Figure 9C_3_). Essentially, this distinction between active waveguide and passive waveguide reflects exciton transfer behavior across materials during excitation [112]. If E_g_‐input > E_g_‐output, excitons can transfer from the input site to the output site and subsequently radiate light, manifesting as an active waveguide. Conversely, when E_g_‐input ≤ E_g_‐output, exciton transfer is hindered, resulting in a passive waveguide.

For example, in a triblock heterostructure, exciting different sites (Input1 and Input2) enables optical logic operations based on the active (artificially defined as “1”) or passive (defined as “0”) waveguide outputs at both tips [90]. Excitation of Input1 gives passive outputs (“0”, “0”) at both tips, and Input2 excitation produces signals “0” and “1” at the respective tips (Figure 9C_1_). For the same nanowire, multiple logic operations can be achieved by illuminating it with light of different wavelengths (e.g., ultraviolet and green light) and encoding the resulting colors displayed by the organic hierarchical structure (Figure 9D) [85]. Simultaneously, lasers of different wavelengths (550/650 nm)can be input to discretize the continuous spatial luminescence signal into different digital logic codes (encoding), with the process of their decoding simulated via simulated oscillography (Figure 9E). In addition, varying excitation location controls signal intensity distribution across terminals, and signal modulation and coding of photonic chips can be realized. For example, the center of the excitation heterostructure chip has the highest intensity in the middle of each end, and the signals on both sides are weaker, corresponding to the code “010” (Figure 9F) [89]. Additionally, logic operations can also be performed based on emissive color [29, 113], polarization angles [46, 114], and other parameters. Besides, multiple parameters (intensity, polarization angle, and wavelength) can be combined into one encoding rule. By assigning distinct definitions to each parameter, the rule follows that: the greater the number of “1” outputs, the darker the color of the corresponding photonic barcodes (Figure 9G). These various operational approaches demonstrate the multifunctionality and flexibility of OHSs, offering broader perspectives for nanophotonic devices.

### 5.7 Challenges and Outlook

**FIGURE 10:**
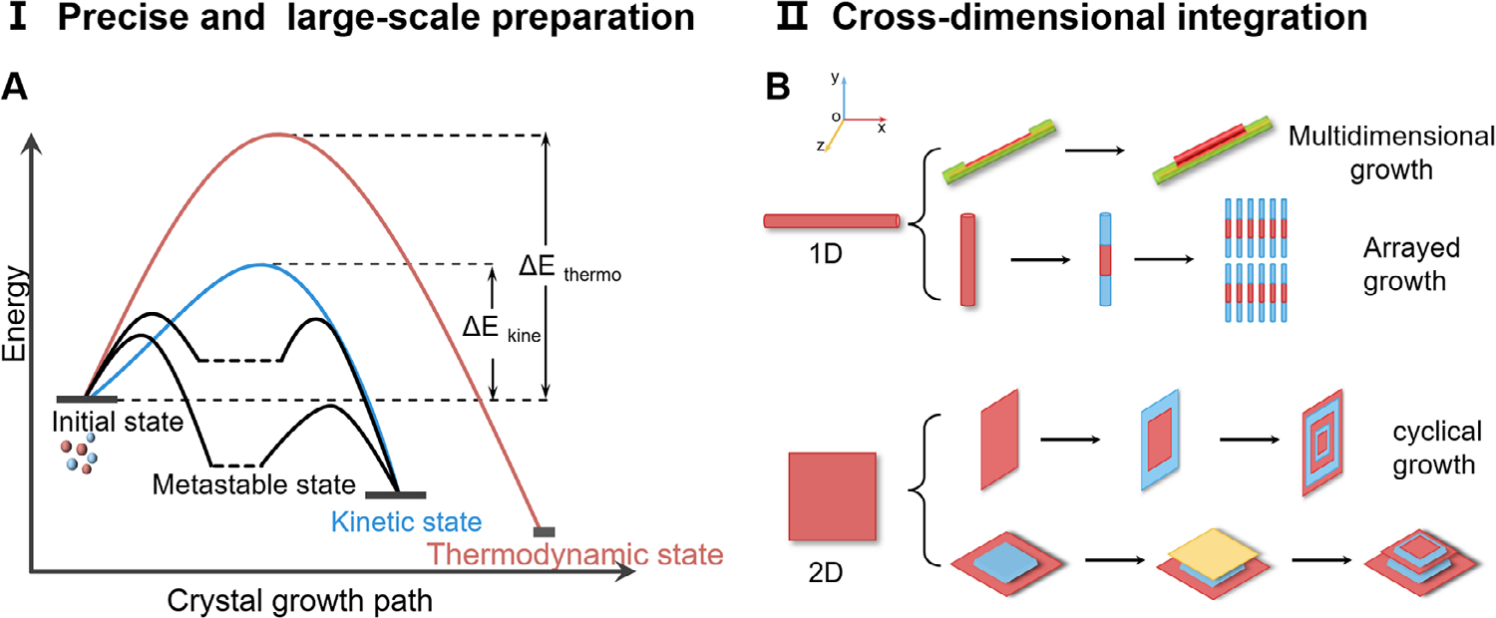
Precise and large‐scale preparation. (A) Balance between thermodynamic stability and kinetic paths. (B) Cross‐dimensional integration.

There are two main challenges for OHSs based on cocrystals. The first one is making precise and large‐scale preparation. By self‐assembly of single cocrystal units with single NCI, is easier to produce large quantities of OHSs with low‐defect density. In contrast, due to the complexity of multiple NCIs in terms of strength and direction, achieving macro‐scale preparation of OHSs is difficult [90]. Furthermore, lattice mismatches of different cocrystals also cannot achieve large‐scale for OHSs. It is well known that during the growth of OHSs, there is a tendency to form thermodynamically stable structures with lower energy/higher energy barriers (Figure 10A). However, certain molecules may lack sufficient energy to form metastable states with higher energy but relative stability [46, 104, 115]. Especially in the process of preparing OHSs using the solvent evaporation method, factors such as solvent evaporation, concentration gradients [75], and environmental temperature [116] can cause branching in the crystal growth pathway, making it difficult to control the uniformity and consistency of the crystal structure, further increasing the complexity of preparation. However, using PVD through a template‐induced approach enables the large‐scale preparation of OHSs. This method still holds considerable potential for future exploration [93].

Another challenge lies in the multi‐dimensional integration and self‐assembly of OHSs. On one hand, the change in the growth direction of OHSs—for instance, a shift from horizontal to vertical orientation, enabling crystals to stand upright—alters the direction of photon/charge transport (Figure 10B). This shortens the transport distance, which is beneficial for enhancing device performance. On the other hand, cross‐dimensional self‐assembly achieves synergistic functionality among multiple components while improving spatial utilization efficiency. This allows for further miniaturization of micro‐optoelectronic devices, resulting in more compact systems and lower costs.

Previously, we have obtained the binding energy between donor and acceptor molecule pairs in cocrystals through DFT calculations, which is crucial for understanding the ordered self‐assembly process. In the future, we could attempt to employ machine learning to screen data such as the sizes of donors & acceptors, lattice parameters, and packing modes of cocrystals. This would help predict candidate cocrystals with well‑matched lattices that are also suitable for large‑scale preparation of OHSs. Furthermore, morphology simulation from Material Studio software could be integrated to analyze the growth direction of OHSs, thereby further evaluating whether the structures of OHSs meet the required specifications. However, achieving precise screening of self‑assembled OHSs will require extensive experimental data and the coupling of multiple computational methods in the early stages. If this vision can be realized in the future, it will represent a key milestone in the on‑demand design and efficient development of organic functional materials. However, to achieve precise screening of self‐assembled targets for OHSs, extensive experimental groundwork and the coupling of multiple computational methods are essential in the early stages. If this vision can be realized in the future, it will represent an essential milestone for tailored design and efficient creation of organic functional materials.

## Conflicts of Interest

The authors declare no conflict of interest.
